# A level-set approach to joint image segmentation and registration with application to CT lung imaging

**DOI:** 10.1016/j.compmedimag.2017.06.003

**Published:** 2018-04

**Authors:** Piotr Swierczynski, Bartłomiej W. Papież, Julia A. Schnabel, Colin Macdonald

**Affiliations:** a Institute for Numerical Mathematics, Technische Universität München, Germany; b Institute of Biomedical Engineering, Department of Engineering Science, University of Oxford, UK; c Division of Imaging Sciences & Biomedical Engineering, King's College London, UK; d Department of Mathematics, The University of British Columbia, Canada

**Keywords:** Joint image segmentation and registration, Atlas-based segmentation, Level-set registration

## Abstract

Automated analysis of structural imaging such as lung Computed Tomography (CT) plays an increasingly important role in medical imaging applications. Despite significant progress in the development of image registration and segmentation methods, lung registration and segmentation remain a challenging task. In this paper, we present a novel image registration and segmentation approach, for which we develop a new mathematical formulation to jointly segment and register three-dimensional lung CT volumes. The new algorithm is based on a level-set formulation, which merges a classic Chan–Vese segmentation with the active dense displacement field estimation. Combining registration with segmentation has two key advantages: it allows to eliminate the problem of initializing surface based segmentation methods, and to incorporate prior knowledge into the registration in a mathematically justified manner, while remaining computationally attractive. We evaluate our framework on a publicly available lung CT data set to demonstrate the properties of the new formulation. The presented results show the improved accuracy for our joint segmentation and registration algorithm when compared to registration and segmentation performed separately.

## Highlights

• A simple novel joint image registration and segmentation method is presented.
• The new algorithm is based on a level-set formulation.
• The algorithm merges Chan–Vese segmentation with active dense displacement estimation.
• Numerical implementation is evaluated on a publicly available lung CT data set.
• Improvement of registration and segmentation properties compared with existing methods is shown.

## 1 Introduction

Image registration and segmentation techniques are fundamental components of medical image analysis as they form the basis for many advanced frameworks for computerized understanding of medical imaging. For example, registration and segmentation of X–ray Computed Tomography (CT) can be used for a vast range of emerging pulmonary applications (Schnabel et al., 2016). Such applications in current and developing medical practice include: personalized adjustment of image-guided radiation therapy (IGRT) (Xing et al., 2006), assessment of disease and treatment progression, e.g. measuring temporal changes of tumor volume (Weiss et al., 2007) or diagnosis of primary pulmonary functions such as assessment of regional ventilation (Guerrero et al., 2005).

Several techniques approaching an automated partitioning of the lungs from CT have been extensively studied for a wide range of clinical pathologies and imaging protocols, and a recent review can be found here (Doel et al., 2015). Volumetric CT scans can be acquired either at different phases of the respiratory cycle (four-dimensional CT), or at different distinctive time points of treatment. Therefore, accurate lung image registration has to be applied in order to provide a common reference space to extract meaningful results for quantitative image analysis. Various registration methods for lung imaging have recently been proposed (Papież et al., 2014, Castillo et al., 2010). In spite of that, registration and segmentation of lung volumes are often inherently linked together: segmentation of the organ of interest can be followed by registration either to find correspondences between consecutive medical volumes acquired during treatment (longitudinal studies) or to compensate for the motion caused by e.g. breathing, or the heart. Whereas segmentation and registration, when performed as separate elements of the processing pipeline, are usually more susceptible to image noise or algorithm initialization, joint segmentation and registration approaches have been shown to be a more appropriate choice when complex medical applications are considered (Yezzi et al., 2001, Gorthi et al., 2011). One of the first attempts to segment the same object in two images, where one image is warped by a deformation was introduced in Yezzi et al. (2001). Paragios et al. (2003) proposed a joint registration and segmentation model using an active contours framework. While in Yezzi et al. (2001) the deformation model was restricted to be rigid, the motion model considered in Paragios et al. (2003) was also able to capture local non-linear deformations. In Vemuri et al. (2003), segmentation-based registration using a level-set approach was proposed. This was extended in Gorthi et al. (2011) to a generalized registration framework: an active deformation field, which merges particularly well different approaches for non-linear contour matching.

In this paper we present a novel approach for joint segmentation and registration using popular level-set algorithms (Tsai et al., 2003; Cremers et al., 2007), which have been specifically adapted to address the issues of separate lung segmentation and registration. The level-set, which is driving the non-linear image registration, is tracked by the dense displacement field similarly as in Vemuri et al. (2003), where the process of matching surfaces was estimated on a voxel-based level. Additionally, the propagation of the surface is extended by a term describing regional properties of the objects of interest similar to classic Chan–Vese segmentation (Chan et al., 2001). Through this, we can include prior information from both dense image intensity features (i.e. intensity values from the CT volumes), and local statistics of the objects of interest to obtain the spatial transformation between images and segmentation. In contrast to similar work on registration and segmentation of lung radiotherapy data (Xue et al., 2010), where a two-step segmentation and registration are iteratively repeated, our method is designed to perform truly joint registration and segmentation by treating both terms within each iteration step. The presented work can also handle segmentation of several 3D objects (in our case left and right lung are segmented separately), what extends some earlier research on joint binary (two objects) segmentation and registration (Unal and Slabaugh, 2008, Le Guyader and Vese, 2011).

The paper is organized as follows: In Section 2, the background on level-set algorithms is briefly presented. Later in this section we also describe the classic Chan–Vese algorithm (Chan et al., 2001) for segmentation of images based on intensities (Section 2.2). Next, we present the state-of-the-art level-set registration algorithm proposed in Vemuri et al. (2003) (Section 2.3) together with its extension to a generalized framework for non-linear level-set registration developed in Gorthi et al. (2011) (Section 2.4). In Section 3 the aforementioned level-set registration and segmentation algorithms are then coupled and form a novel joint registration and segmentation method, merging algorithms previously proposed separately for segmentation (Chan et al., 2001) and for registration (Vemuri et al., 2003). We also describe the details of the numerical implementation of our method in Section 4. Our new algorithm is compared against the state-of-the-art algorithms presented in this paper (Vemuri et al., 2003, Chan et al., 2001), and the results of this evaluation are presented in Section 5. The evaluation is performed using a publicly available lung CT data set (*Dir-Lab*) (Castillo et al., 2009) and assessed using the Dice overlap measure and the Target Registration Error (TRE) as segmentation and registration accuracy estimates, respectively. Finally, the paper is concluded in Section 6.

## 2 Background

### 2.1 Level-set methods

Level-set methods, originally introduced by Osher and Sethian (1988), provide a very effective framework for numerical description of curves and surfaces and therefore are widely applicable in many areas including computational fluid dynamics problems (Sussman and Fatemi, 1999, Tryggvason et al., 2001), as well as image processing and computer vision applications (Chan et al., 2000, Vese and Chan, 2002, Zhao et al., 2000).

The principal idea behind level-set methods is to avoid explicit parametric representation of geometrical objects such as curves or surfaces, and instead represent these objects implicitly in terms of a function defined on a fixed computational grid. Contrary to explicit contour representations, level-set methods are also successful in capturing topological changes of objects. For example, level-set can easily handle splitting of a connected region into two or more disjoint parts (Osher and Fedkiw, 2006).

Curves and surfaces can be described implicitly as the zero level-sets of some sufficiently smooth function *ϕ*:

(1) C={x∈Ω:ϕ(x)=0}

Here **x** denotes a point in a region Ω.

### 2.2 Level-sets for segmentation

Chan et al. (2001) proposed an algorithm, which has since been widely used for different image segmentation tasks (Osher and Fedkiw, 2006, Cremers et al., 2007), including medical images (see e.g., Tsai et al., 2003; Paragios, 2003). It is a special case of the Mumford–Shah optimal partition and approximation problem (Mumford and Shah, 1989) designed for binary images. However, it also gives very good results in case of gray-scale and vector-valued (e.g., RGB) images (Chan et al., 2000). Next, we briefly review the method. Suppose we are given a domain Ω divided by a contour Γ = {*ϕ*(**x**) = 0} into two (possibly unconnected) subregions Ω_in_ = {*ϕ*(**x**) > 0} and Ω_out_ = {*ϕ*(**x**) < 0}. The function *ϕ* is a level-set function defining the segmenting contour. Let *I*(**x**) be an image defined on the region Ω. The method relies on the minimization of an intensity-based energy functional given by:

(2) $$E(\phi ,c_{1},c_{2})=|\Gamma |+\int_{\Omega_{\text{in}}}|I(\text{x})-c_{1}{|}^{2}d\text{x}+\int_{\Omega_{\text{out}}}|I(\text{x})-c_{2}{|}^{2}d\text{x},$$

where |Γ| is a length of the segmenting contour, and *c*_1_ and *c*_2_ denote average intensities inside and outside of the segmenting contour Γ, respectively, in the following way:

(3) $$c_{1}=\frac{1}{\left|\Omega_{\text{in}}\right|}\int_{\Omega_{\text{in}}}I(\text{x})\;d\text{x},\;c_{2}=\frac{1}{\left|\Omega_{\text{out}}\right|}\int_{\Omega_{\text{out}}}I(\text{x})\;d\text{x}.$$

Hence, the functional *E* given in (2) penalizes local discrepancy from the average intensity in the segmented regions. Using the gradient flow method (Ambrosio et al., 2008), the regularized minimization problem can be turned into an evolutionary partial differential equation (PDE) on the function *ϕ* (Chan et al., 2001).

The Chan–Vese algorithm is robust with respect to noise (Chan et al., 2001) and as such it can be applied to medical images containing inevitable acquisition artefacts. Additionally, it can successfully segment images without large intensity gradients, i.e., without sharp edges. It is worth noting that the Chan–Vese algorithm can be extended to vector-valued images (Chan et al., 2000) and to finding several disjoint regions at the same time (Vese and Chan, 2002).

### 2.3 Level-sets for registration

Suppose we are given two images, the source *I*_*S*_ and the target (reference) *I*_*T*_, defined on a rectangular domain $\Omega \subset {\mathbb{R}}^{n}$, where *n* is either 2 or 3. Level-set based image registration algorithm proposed in Vemuri et al. (2003) (referred to later as Vemuri's algorithm) was designed as a minimization of a difference between the input images *I*_*S*_ and *I*_*T*_, as measured in the *L*^2^-norm. This is achieved by transforming the source image *I*_*S*_ directly onto *I*_*T*_ by evolving according to

(4) $$\frac{\partial J}{\partial t}=(I_{T}-I_{S})|\nabla J|,\;J(\text{x},0)=I_{S}(\text{x}),$$

where (*I*_*T*_ − *I*_*S*_) can be considered as a level-set velocity function evolving along the gradient ∇*J*. This registration algorithm can be understood in terms of a level-set framework as matching the intensity contours of images *I*_*S*_ and *I*_*T*_. However, the final result of the registration algorithm that we are looking for is a plausible displacement field $\text{u}(\text{x}):{\mathbb{R}}^{n}\to {\mathbb{R}}^{n}$ such that images *I*_*T*_(**x**) and *I*_*S*_(**x** + **u**(**x**)) are similar in some sense. Supposing that **u** depends on the artificial iteration time *t* and according to (4), we can obtain that the displacement vector field **u**(**x**) as a limit *t* → ∞ of **u**(**x**, *t*). When **u** is the solution of the evolution equation:

(5) $$\frac{\partial \text{u}}{\partial t}=\left(I_{T}(\text{x})-I_{S}(\text{U}(\text{x},t))\right)\frac{\nabla I_{S}(\text{U}(\text{x},t))}{\left|\nabla I_{S}(\text{U}(\text{x},t)))\right|}=:\text{Vem}(\text{x},t),$$

where **U(**x****, *t*) = **x** + **u**(**x**, *t*). We use **u**(**x**, 0) = **0** as an initial condition for the problem. Since the gradient calculation in Eq. (5) is sensitive to noise, the input images are usually smoothed with a Gaussian kernel of variance *σ*_1_ as a preprocessing step.

### 2.4 Joint registration and segmentation framework

Vemuri's level-set registration algorithm (Vemuri et al., 2003) is a purely intensity-based method. It does not take into consideration any prior knowledge about anatomical structures or physiological features apparent in the registered images. However, incorporating some prior knowledge about them has been shown to improve the accuracy of the registration (Yezzi et al., 2001).

Gorthi et al. (2011) generalized the approach used in Vemuri's algorithm. Let *ϕ*_*G*_ be a level-set function. Similar reasoning as in the case of Vemuri's algorithm leads to an evolutionary equation defining a way of finding the displacement field **u**(**x**, *t*)

(6) $$\frac{\partial \text{u}}{\partial t}=\beta \left(\phi_{G}(\text{U}(\text{x},t))\right)\frac{\nabla \phi_{G}(\text{U}(\text{x},t))}{\left|\nabla \phi_{G}(\text{U}(\text{x},t))\right|},$$

subject to **u**(**x**, 0) = **0**. Here *β*(*ϕ*_*G*_) is a velocity function characterizing the model. The final displacement field **u**(**x**) is then obtained as a limit $\text{u}(\text{x})=\lim_{t\to \infty }\text{u}(\text{x},t)$. Notice that Eq. (5) is a special case of Eq. (6), for the intensity function being chosen as a level-set function and subject to the suitable choice of the velocity *β*. This approach allows for various choices of the function *ϕ*_*G*_ and hence, a wide scope of prior knowledge about geometrical (and anatomical) features can be easily incorporated into the model. Moreover, there exists a freedom of choice of the velocity function *β*. Finally, several methods can be combined and different types of forces, even working on different level-sets, can be taken into consideration at the same time by adding them to the right hand side of Eq. (6).

Gorthi et al. (2011) proposed also a particular choice of the level set function *ϕ*_*G*_ and velocity *β* resulting in an algorithm being an example of atlas-based registration. It assumes that the target image *I*_*T*_ can be initially segmented (or there is an atlas available) and distinct regions are labeled. This initial segmentation can be done either manually by an expert or using an automated segmentation algorithm such as the one proposed in Section 2.2. Then, the spatial transformation between the registered image *I*_*S*_ and the target image *I*_*T*_ is estimated by exploiting this prior segmentation as an additional information to drive the registration. Finally, the labels from the initial segmentation in the target image regions are propagated onto the source image using the estimated displacement and therefore the segmentation of the source image is obtained. This method provides not only a spatial correspondence between two images but also allows the segmentation of several objects in a given image at the same time. The accuracy of the method depends both on the accuracy of the prior segmentation and the registration algorithm. This approach has been successfully applied in medical imaging due to the ability of exploiting prior knowledge about the anatomical structures.

Suppose that the segmentation of the region Ω is given and that Ω can be written as a union of *k* non-overlapping subregions, i.e. Ω = ⋃ Ω_k_. Consider a level-set function defining this segmentation:

(7) $$\phi_{L}(\text{x})=k,\;\text{when}\;\text{x}\in \Omega_{k}.$$

**Fig. 1:**
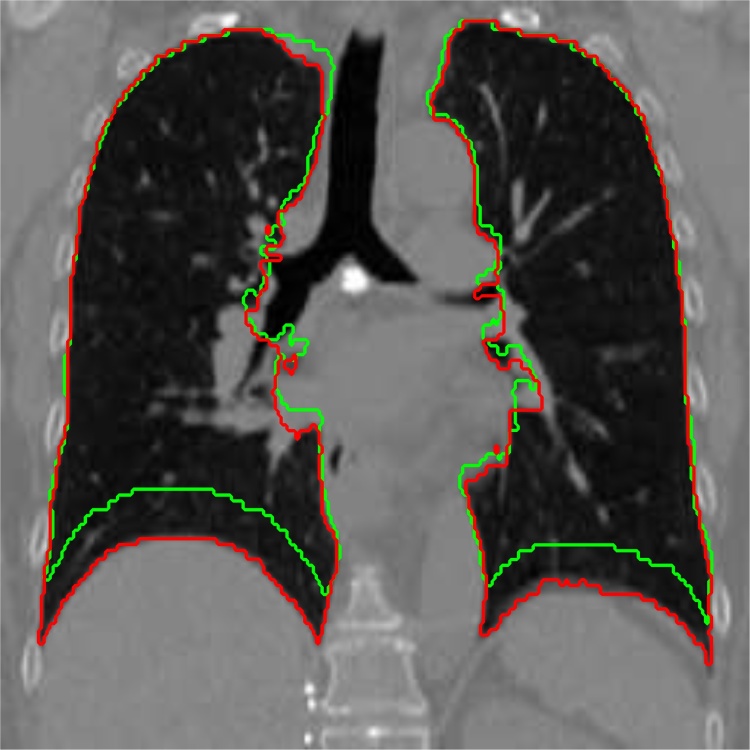
Example of superimposed lung segmentations in inhale state (red) and exhale state (green). Segmentation was performed by experts. The image comes from publicly available *Dir-Lab* set of CT data described in Castillo et al. (2009). Function *ϕ*_*L*_ introduced in Eq. (7) takes value 1 inside the red contour and value 0 outside of it. (For interpretation of the references to color in this figure legend, the reader is referred to the web version of this article.)

Because in this work we focus on lung images, we shall assume that Ω is divided into sets Ω_in_ representing the lungs and Ω_out_ representing outer parts of the thoracic cage, see Fig. 1.

Although the function *ϕ*_*L*_ captures geometrical information about the shapes in the image, it cannot be directly used in Eq. (6) due to the jump discontinuity between regions meaning that the gradient of function *ϕ*_*L*_ is not well-defined. To avoid this problem, some regularization by a convolution function *ϕ*_*L*_ with a Gaussian kernel *G*_*σ*_ of variance *σ* is added. Notice that *G*_*σ*_ * *ϕ*_*L*_ ∈ *C*^∞^(Ω). Moreover, the geometrical description of the boundaries of sets Ω_*k*_ is preserved. However, now these are no longer modeled by discontinuities in the level-set function but by the local maximum of the magnitude of the gradient of the regularized level-set function. It is also worth noting that convolution with the Gaussian kernel has a denoising effect on the image *I*_*S*_ as it does in Vemuri's force (given by Eq. (5)).

In principle, the direction of the evolution of the displacement field is controlled by the gradient of the regularized level-set function together with the velocity term *β*. However, following (Gorthi et al., 2011) a modified sign function *S*(**x**) : Ω → { −1, 0, 1} is used so that the vector *S*(*x*) ∇ (*G*_*σ*_ * *ϕ*_*L*_) is always pointing from Ω_in_ to Ω_out_. This function is well defined and nonzero in narrow bands around region boundaries and set to 0 outside those bands.

To complete the definition of the velocity *β* from Eq. (6), we set *c*_1_ and *c*_2_ to be the mean intensities of the image *I*_*T*_ in the region Ω_in_ and Ω_out_, respectively as in Eq. (3). These values are kept constant throughout the registration process and are computed once at the beginning. We choose the evolution velocity to be defined as follows:

(8) $$\beta (\text{x},t)=-S(\text{x})\left({\left(I_{S}(\text{U}(\text{x},t))-c_{\text{in}}\right)}^{2}-{\left(I_{S}(\text{U}(\text{x},t))-c_{\text{out}}\right)}^{2}\right).$$

This velocity term is inspired by the forces used in the Chan–Vese segmentation algorithm presented in Section 2.2.

Here we assume that the regions that are to be segmented in target and register source images, have the same intensities. Distances between image intensities can be reduced by matching the histograms prior to the registration algorithm.

Finally, the evolution of Eq. (6) takes the form

(9) $$\frac{\partial \text{u}}{\partial t}={\text{GCV}}_{1}(\text{x},t),$$

subject to **u**(**x**, 0) = **0**, where

(10) $${\text{GCV}}_{1}(\text{x},t)=\beta (\text{x},t)\frac{\nabla \left(\left(G_{\sigma }*\phi_{L})(\text{U}(\text{x},t)\right)\right)}{\left|\nabla \left(\left(G_{\sigma }*\phi_{L})(\text{U}(\text{x},t)\right)\right)\right|}.$$

Note that the forces in Eq. (9) have a very local behavior due to the choice of the special sign function *S* in Eq. (8), being zero far from contours segmenting the image *I*_*T*_. This means that the points lying outside narrow bands surrounding these contours are not undergoing deformation in time. To propagate the information from the segmenting contour, we add an additional diffusion term to the evolution equation. Let ∇^2^**u** denote the spatial Laplace operator acting on each of the vector components of the displacement field separately. Letting *ϵ* be a small positive parameter, (9) can be modified to in the following way:

(11) $$\frac{\partial \text{u}}{\partial t}={\text{GCV}}_{1}(\text{x},t)+\epsilon \nabla^{2}\text{u}(\text{x},t),$$

subject to **u**(**x**, 0) = **0**. Note that the Laplacian of the deformation field **u**(**x**, *t*) is taken component wise. The primary role of the diffusion term ∇^2^**u** is to propagate information coming from the mentioned narrow band over the whole domain Ω (Eq. (11) refers to voxelwise representation of the contour only). Nevertheless, it acts also as an additional regularization, smoothing the evolving displacement field **u** (Modersitzki, 2009), which is a representation of contour in our formulation of level-sets.

A huge variety of the regularizing terms for deformable image registration has been considered in image processing applications, many being modifications of the heat-equation approach presented here. Among them there are anisotropic diffusion filtering methods described in Weickert (1997). Related to that is a bilateral filtering method that can be used to capture the sliding motion between organs, occurring for example between lungs and liver or between lung lobes (Papież et al., 2014). The framework described here is flexible enough to take these into account.

## 3 The new model description

The framework developed in Gorthi et al. (2011) enables the simultaneous use of several types of level-set functions. This can be achieved by modifying forces on the right hand side of Eq. (11).

So far we considered the segmentation of the target image *I*_*T*_ as dividing it into two regions. However, this approach can be generalized to an arbitrary number of subregions of Ω and Eq. (11) can be adjusted accordingly. Suppose now that the domain Ω is split into subregions $\Omega_{\text{in}}^{1}$, $\Omega_{\text{in}}^{2}$, and $\Omega_{\text{out}}^{1}$, $\Omega_{\text{out}}^{2}$, respectively. Moreover, assume that the sets $\Omega_{\text{in}}^{1}$, $\Omega_{\text{in}}^{2}$ are strongly disjoint. By this we mean that $\text{dist}(\Omega_{\text{in}}^{1},\Omega_{\text{in}}^{2})>0$. The motivation for considering this case is the application to lung scans in which each lung is segmented separately. We can redefine forces used for determining the displacement field **u** in a straightforward manner. We replace the averages *c*_in_ and *c*_out_ with the average intensities $c_{\text{in}}^{1}$, $c_{\text{in}}^{2}$, $c_{\text{out}}^{1}$, $c_{\text{out}}^{2}$ taken over newly segmented regions. By changing the velocity function *β*, we replace the operator GCV_1_ with its extended version being defined as follows:

(12) $$\begin{array}{cc} {\text{GCV}}_{2}(\text{x},t)= & -S(\text{x})\left[{\left(I(\text{U}(\text{x},t))-c_{\text{in}}^{1}\right)}^{2}-{\left(I(\text{U}(\text{x},t))-c_{\text{out}}^{1}\right)}^{2}\right)\\ & \left(+{\left(I(\text{U}(\text{x},t))-c_{\text{in}}^{2}\right)}^{2}-{\left(I(\text{U}(\text{x},t))-c_{\text{out}}^{2}\right)}^{2}\right]\\ & \frac{\nabla \left(\left(G_{\sigma_{2}}*\phi_{L})(\text{U}(\text{x},t)\right)\right)}{\left|\nabla \left(\left(G_{\sigma_{2}}*\phi_{L})(\text{U}(\text{x},t)\right)\right)\right|}. \end{array}$$

We propose one more extension of Gorthi's algorithm (Gorthi et al., 2011) by taking into account several regions segmented independently. This extension is kept in the spirit of Gorthi's algorithm but more than one level-set function of the kind given in (7) is used. We take $\phi_{L}^{1}(\text{x})=\chi_{\Omega_{\text{in}}^{1}}(\text{x})$ and $\phi_{L}^{2}(\text{x})=\chi_{\Omega_{\text{in}}^{2}}(\text{x})$, so the level-set functions $\phi_{L}^{1}$ and $\phi_{L}^{2}$ are defined as characteristic functions of regions $\Omega_{\text{in}}^{1}$ and $\Omega_{\text{in}}^{2}$ respectively. We define a new vector field governing evolution of the displacement field as follows:

(13) $$\begin{array}{c} {\text{GCV}}_{3}(\text{x},t)=\\ -S_{1}(\text{x})\left[{\left(I(\text{U}(\text{x},t))-c_{\text{in}}^{1}\right)}^{2}-{\left(I(\text{U}(\text{x},t))-c_{\text{out}}^{1}\right)}^{2}\right]\frac{\nabla \left(\left(G_{\sigma_{2}}*\phi_{L}^{1})(\text{U}(\text{x},t))\right)\right)}{\left|\nabla \left(\left(G_{\sigma_{2}}*\phi_{L}^{1})(\text{U}(\text{x},t)\right)\right)\right|}\\ -S_{2}(\text{x})\left[{\left(I(\text{U}(\text{x},t))-c_{\text{in}}^{2}\right)}^{2}-{\left(I(\text{U}(\text{x},t))-c_{\text{out}}^{2}\right)}^{2}\right]\frac{\nabla \left(\left(G_{\sigma_{2}}*\phi_{L}^{2})(\text{U}(\text{x},t)\right)\right)}{\left|\nabla \left(\left(G_{\sigma_{2}}*\phi_{L}^{2})(\text{U}(\text{x},t)\right)\right)\right|}, \end{array}$$

Because the sets $\Omega_{\text{in}}^{1}$ and $\Omega_{\text{in}}^{2}$ are strongly disjoint by assumption and since *ϕ*_*L*_ is independent of time, we can always find a strictly positive parameter *γ* such that $\text{dist}(\Omega_{\text{in}}^{1},\Omega_{\text{in}}^{2})>2\gamma$. By setting the sign functions *S*_1_ and *S*_2_ to zero when **x** does not belong to a band of width *γ* around $\partial \Omega_{\text{in}}^{1}$ and $\partial \Omega_{\text{in}}^{2}$ respectively, we ensure that two elements of the vector field given in Eq. (13) have no influence on each other. Moreover, *S*_1_ and *S*_2_ are equal to 1 otherwise since the numerical values of level-set functions $\phi_{L}^{1}$, $\phi_{L}^{2}$ are assigned suitably by construction.

The evolution problem for registration using prior segmentation of the selected regions can be defined by replacing GCV_1_ with the operator GCV_2_ or GCV_3_ in (11).

As pointed out above, many evolution forces can be incorporated in the evolution equation using Gorthi's framework. Moreover, several different level-set functions can be used in Eq. (6) at the same time. In Section 2.3 we noticed that the image intensity function is a valid choice for the level-set function. We propose a novel combination of forces using modifications of the evolution equation proposed by Gorthi et al. (2011) together with Vemuri's registration method.

Let *θ* ∈ [0, 1] be a weighting parameter. Consider the displacement field evolution problem

(14) $$\frac{\partial \text{u}}{\partial t}=\theta {\text{GCV}}_{j}(\text{x},t)+(1-\theta )\text{Vem}(\text{x},t)+\epsilon \nabla^{2}\text{u},$$

subject to **u**(**x**, 0) = **0** and *j* ∈ {1, 2, 3}. The evolution problem given in Eq. (14) defines a joint segmentation and registration method combining the approaches proposed by Vemuri et al. (2003) and Gorthi et al. (2011). Note that each of them can be recovered when we choose *θ* = 0 and *θ* = 1 respectively. When *θ* ∈ (0, 1), this method should bring advantages of both methods together. It exploits the prior geometrical or anatomical knowledge about regions in the images. Moreover, it uses the intensity function for matching the regions, so that the registration acts on the entire image, rather than only relying on the level-set propagation by additional diffusion filtering.

To obtain a segmentation of the image *I*_*S*_, we first label the regions Ω_in_ and Ω_out_ by assigning the values 1 and 0 to the points in these regions. These labels are transferred to the image *I*_*S*_ by adding the displacement field

(15) $$\begin{array}{c} \Omega_{\text{in}}^{I}=\left\{\text{x}:\;\text{x}=\text{y}+\text{u}(\text{y}),\;\text{y}\in \Omega_{\text{in}}\right\},\\ \Omega_{\text{out}}^{I}=\Omega \backslash \Omega_{\text{in}}^{I}. \end{array}$$

A similar procedure is used when the image *I*_*T*_ is divided into more than two regions by increasing the number of labels accordingly.

## 4 Numerical implementation

In this work we deal with images which are normalized (prior to segmentation and registration) to take values from the interval [0, 1].

The Chan–Vese segmentation algorithm presented in Section 2.2 was implemented as proposed in Chan et al. (2001). In the numerical implementation of the registration methods described in Section 2, we use a finite difference discretization for numerical differentiation and a forward Euler scheme for numerical time integration. Since this time-stepping scheme may lead to instabilities in the solution, a time step of size Δ*t* = 0.5 was empirically chosen. In the numerical implementation of algorithms presented in this article, we use the grid naturally defined by the image voxels. That is, we choose constant step-sizes *h*_*x*_ = *h*_*y*_ = *h*_*z*_ = 1 in each direction, so that the mesh is given by **x**_*i*,*j*,*k*_ = (*x*_*i*_, *y*_*j*_, *z*_*k*_) ∈ Ω, 1 ≤ *i* ≤ *M*_*x*_, 1 ≤ *j* ≤ *M*_*y*_ and 1 ≤ *k* ≤ *M*_*z*_. The region Ω is assumed to be a cuboid with respective edges of lengths *M*_*x*_, *M*_*y*_ and *M*_*z*_.

We define the finite differences of a function *ϕ* by

(16) $$\begin{array}{c} D_{x}^{-}\phi_{i,j,k}=\frac{\phi_{i,j,k}-\phi_{i-1,j,k}}{h_{x}},\;D_{x}^{+}\phi_{i,j,k}=\frac{\phi_{i+1,j,k}-\phi_{i,j,k}}{h_{x}},\\ D_{y}^{-}\phi_{i,j,k}=\frac{\phi_{i,j,k}-\phi_{i,j-1,k}}{h_{y}},\;D_{y}^{+}\phi_{i,j,k}=\frac{\phi_{i,j+1,k}-\phi_{i,j,k}}{h_{y}},\\ D_{z}^{-}\phi_{i,j,k}=\frac{\phi_{i,j,k}-\phi_{i,j,k-1}}{h_{z}},\;D_{z}^{+}\phi_{i,j,k}=\frac{\phi_{i,j,k+1}-\phi_{i,j,k}}{h_{z}}. \end{array}$$

We call *D*^−^, *D*^+^ the backward and forward difference respectively.

To improve the numerical stability of this scheme, we replace the Euclidean norm |v| in Eq. (14) with $|v{|}_{\alpha }=\sqrt{|v{|}^{2}+\alpha^{2}}$, where *α* is a small parameter (*α* ≈ 10^−4^). We set ${\text{u}}_{i,j,k}^{n}=\text{u}(x_{i},y_{j},z_{k},n\Delta t)$. Let us also define *ϕ*_*L*;*i*,*j*,*k*_ = *ϕ*_*L*_(**x**_*i*,*j*,*k*_), $I_{S;i,j,k}^{n}=I_{S}({\text{x}}_{i,j,k}+{\text{u}}_{i,j,k}^{n})$, *I*_*T*;*i*,*j*,*k*_ = *I*_*T*_(**x**_*i*,*j*,*k*_) to be images discretized on a given mesh and

(17) $${\left(C_{\phi_{G}}\right)}_{i,j,k}^{n}=G_{\sigma }*\phi_{G}({\text{x}}_{i,j,k},n\Delta t)$$

be a smoothed general level-set function. Note that replacing *ϕ*_*G*_ by *ϕ*_*L*_ we obtain $C_{\phi_{L}}$, which is independent of time. In contrast, $C_{S}=G_{\sigma }*I_{S;i,j,k}^{n}$ evolves in time. In evaluating $I_{S;i,j,k}^{n}$, linear interpolation in neighbouring points of ${\text{x}}_{i,j,k}+{\text{u}}_{i,j,k}^{n}$ is used.

The gradient of the level-set function and its norm are approximated with

(18) $$\begin{array}{cc} \nabla & \left(G_{\sigma }*\phi_{G}({\text{x}}_{i,j,k},n\Delta t)\right)\\ & \approx \left(\begin{array}{c} m(D_{x}^{+}{\left(C_{\phi_{G}}\right)}_{i,j,k}^{n},D_{x}^{-}{\left(C_{\phi_{G}}\right)}_{i,j,k}^{n})\\ m(D_{y}^{+}{\left(C_{\phi_{G}}\right)}_{i,j,k}^{n},D_{y}^{-}{\left(C_{\phi_{G}}\right)}_{i,j,k}^{n})\\ m(D_{z}^{+}{\left(C_{\phi_{G}}\right)}_{i,j,k}^{n},D_{z}^{-}{\left(C_{\phi_{G}}\right)}_{i,j,k}^{n}) \end{array}\right)=:{\left(\nabla \phi_{G}\right)}_{i,j,k}^{n} \end{array}$$

and

(19) $$\begin{array}{c} |\nabla (G_{\sigma }*\phi_{G}({\text{x}}_{i,j,k},n\Delta t)){|}_{\alpha }\approx |{\left(\nabla \phi_{G}\right)}_{i,j,k}^{n}{|}_{\alpha }\\ ≔\sqrt{\alpha^{2}+\sum_{s\in \{x,y,z\}}{\left(m(D_{s}^{+}{\left(C_{\phi_{G}}\right)}_{i,j,k}^{n},D_{s}^{-}{\left(C_{\phi_{G}}\right)}_{i,j,k}^{n})\right)}^{2}}, \end{array}$$

where following the implementation presented in Vemuri et al. (2003) we use the *minmod finite difference* scheme (Osher and Sethian, 1988) with

(20) $$m(x,y)=\left\{\begin{array}{cc} \text{sign}(x)\min(|x|,|y|), & \;x\cdot y\geq 0\\ 0, & \;\text{otherwise}. \end{array}\right)$$

We obtain a discretization of the Vemuri's force term (5)

(21) $${\text{Vem}}_{i,j,k}^{n}≔\left(I_{T;i,j,k}-I_{S;i,j,k}^{n}\right)\frac{{\left(\nabla I\right)}_{S;i,j,k}^{n}}{|{\left(\nabla I\right)}_{S;i,j,k}^{n}{|}_{\alpha }}.$$

We approximate the mean intensities for (3) of the target image *I*_*T*_ used in the definitions of operators GCV_*j*_ with mean intensities computed by

(22) $$\begin{array}{c} c_{\text{in}}\approx {\tilde{c}}_{\mathrm{in}}≔\frac{1}{\#\{{\text{x}}_{i,j,k}\in \Omega_{\text{in}}\}}\sum_{i,j,k:\;{\text{x}}_{i,j,k}\in \Omega_{\text{in}}}I_{T;i,j,k},\\ c_{\text{out}}\approx {\tilde{c}}_{\mathrm{out}}≔\frac{1}{\#\{{\text{x}}_{i,j,k}\in \Omega_{\text{out}}\}}\sum_{i,j,k:\;{\text{x}}_{i,j,k}\in \Omega_{\text{out}}}I_{T;i,j,k}, \end{array}$$

where #X denotes the number of elements of the set X. Hence, the operator GCV_1_ defined in Eq. (10) is discretized by taking

(23) $$\begin{array}{cc} {\text{GCV}}_{1}({\text{x}}_{i,j,k},n & \Delta t)\approx {\text{Gor}}_{1;i,j,k}^{n}\\ & ≔-S({\text{x}}_{i,j,k})\left[{\left(I_{S;i,j,k}^{n}-{\tilde{c}}_{\text{in}}\right)}^{2}-\right)\\ & \left({\left(I_{S;i,j,k}^{n}-{\tilde{c}}_{\text{out}}\right)}^{2}\right]\frac{{\left(\nabla \phi_{L}\right)}_{i,j,k}^{n}}{|{\left(\nabla \phi_{L}\right)}_{i,j,k}^{n}{|}_{\alpha }}, \end{array}$$

where Gor stands for Gorthi and we follow a discretization introduced in Gorthi et al. (2011). Operators GCV_2_ and GCV_3_ are approximated in a similar way. Moreover, in the definitions (12) and (13) we choose $\Omega_{\text{out}}^{1}=\Omega_{\text{out}}^{2}$ to represent the part of the image not occupied by lungs.

In the numerical implementation we split each time step into two stages. The first stage neglects the diffusion term: by combining Eq. (23), Eq. (21) and Eq. (14) with *ϵ* = 0 and discretizing in time with Forward Euler scheme we obtain

(24) $$\begin{array}{c} {\tilde{\text{u}}}_{i,j,k}^{n+1}={\text{u}}_{i,j,k}^{n}+\Delta t\;\left[\theta {\text{Gor}}_{m;i,j,k}^{n}+(1-\theta ){\text{Vem}}_{i,j,k}^{n}\right]\\ \text{with}\;{\text{u}}_{i,j,k}^{0}=0\;\text{and}\;m\in \{1,2,3\}. \end{array}$$

The second stage solves the diffusion equation $\frac{\partial \text{u}}{\partial t}=\Delta \text{u}$ for a small time by convolving the numerical solution ${\tilde{\text{u}}}_{i,j,k}^{n+1}$ with a Gaussian kernel $G_{\sigma_{3}}$ of a variance *σ*_3_:

(25) $${\text{u}}_{i,j,k}^{n+1}=G_{\sigma_{3}}*{\tilde{\text{u}}}_{i,j,k}^{n+1}.$$

Note that the choice of parameter *σ*_3_ depends on the values of *ϵ*, *θ* and Δ*t*. In the numerical tests, in which we use Δ*t* = 0.5, *ϵ* = 1 and *θ* = 0.5 or *θ* = 1, the variance *σ*_3_ equal to the size of two voxels appears to be a good choice. Note that when we take *θ* = 0 (and so recover Vemuri's algorithm as in Eq. (14)), there is no need for including the extra diffusion term and we can skip the second stage.

To obtain a segmentation of the image *I*_*S*_, we first label the regions Ω_in_ and Ω_out_ by assigning the values 1 and 0 to the voxels in these regions. These labels are then transferred to the image *I*_*S*_. A similar procedure is used when the image *I*_*T*_ is divided into more than two regions by increasing the number of labels accordingly.

The 3D algorithm was applied to real human CT lung images of the resolution of at least 256 × 256 × 108 consisting of more than 7 million voxels. To reduce the computational cost of the method, we use a multi-resolution approach. First, the algorithm is performed on an image of quarter-resolution in each dimension, (thus 64 × 64 × 27 instead of 256 × 256 × 108). These are obtained by image convolution with a step function *χ*_[−2,2]×[−2,2]×[−2,2]_, the characteristic function of the cube [−2, 2] × [−2, 2] × [−2, 2] and sampling the averaged image at the coarser grid. The segmentation obtained at this level is then used as an initial condition for the calculation at the next level. The next level consists of an image obtained similarly but with half-resolution in each direction (128 × 128 × 54). Finally, the result from this middle-resolution level is used as an initial condition for the registration of the original image.

Numerical tests confirm that the multi-resolution approach gives comparable results to a direct one, but uses fewer iterations overall and significantly decreases the computational cost.

The segmentations (labels) in the data need to be initialized by the user before optimization, and these segmentations can be generated either manually or from human body imaging atlases.

## 5 Experiments and results

In this chapter, we present a comparison of various methods that we have described in previous sections and assess them for joint segmentation and registration of lung CT images. For the evaluation of each method's accuracy we use the publicly available *Dir-Lab* set of CT data described in Castillo et al. (2009). This data set contains 10 pairs of complete 4D CT lung scans of patients suffering from lung or esophageal cancer. The spatial resolution of the data is known and one voxel in the image corresponds to a cuboid of size varying from 0.97 mm × 0.97 mm × 2.5 mm to 1.16 mm × 1.16 mm × 2.5 mm depending on the case. Moreover, each pair of CT images is accompanied by a set of 300 well-distributed landmarks manually identified by experts with the intra-observer error approximately equal to 1.0 mm (Castillo et al., 2009). These landmarks are used to measure the distance between images before and after the registration. Additionally, we used the expert lung segmentations which we will consider to be the gold standard for segmentation assessment.

### 5.1 Segmentation evaluation

To evaluate the accuracy of the segmentation method, we follow a standard approach used in biomedical imaging applications by comparing the Dice measure (Zijdenbos et al., 1994) between segmentation result produced by the assessed algorithms and the expert segmentation. Suppose that the domain Ω is divided into two regions Ω_in_ and Ω_out_. These sets are approximated using a segmentation algorithm by ${\tilde{\Omega }}_{\text{in}}$ and ${\tilde{\Omega }}_{\text{out}}$. Assuming that the set Ω_in_ denotes the region of our interest, for example the lungs, we define the Dice coefficient as follows:

$$\text{Dice}(\Omega_{\text{in}},{\tilde{\Omega }}_{\text{in}})=2\frac{\left|\Omega_{\text{in}}\cap {\tilde{\Omega }}_{\text{in}}\right|}{\left|\Omega_{\text{in}}|+|{\tilde{\Omega }}_{\text{in}}\right|}.$$

**Fig. 2:**
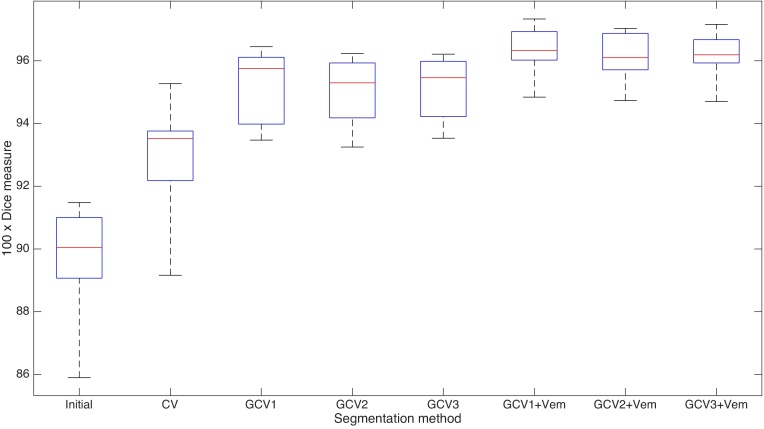
Comparison of Dice coefficients for the segmentation algorithms. CV denotes the Chan–Vese algorithm, GCV_*i*_ represents consecutive modifications of Gorthi's algorithm and GCV_*i*_ + Vem are our coupled Gorthi's–Vemuri methods. Box edges represent 25th and 75th percentiles, the central mark represents the median, and whiskers extend between maximum and minimum values. Our proposed joint registration and segmentation algorithms are more accurate. Gorthi's algorithm gives better results when coupled with Vemuri's method.

Volumes of all regions are approximated by the number of corresponding unit voxels in the images. In our experiments we use the expert segmentation of the lungs in the inhale stage for the *Dir-Lab* database for the sets Ω_in_. The sets ${\tilde{\Omega }}_{\text{in}}$ were approximated using segmentation methods presented before: Chan–Vese algorithm (**CV**), Gorthi's algorithm and its modifications using vector fields Eq. (10), (12), (13) (GCV_1_, GCV_2_ and GCV_3_ respectively) and the joint Gorthi–Vemuri algorithm with *θ* = 0.5 and similar modifications (GCV_1_ + Vem, GCV_2_ + Vem and GCV_3_ + Vem respectively). The complete comparison is summarized in Fig. 2. Segmentation of the lungs in the exhale stage of low resolution 64 × 64 × 27 was used as an initial contour for the Chan–Vese algorithm. The Dice coefficient computed for the initial contour is shown on the left-hand side of Fig. 2. Even though the initial condition used for segmentation overlaps strongly with the region to be segmented, the Chan–Vese algorithm barely improves the results. Notice that the results obtained using registration-based segmentation are better in all of the studied cases. Moreover, as we shall see later, in these cases the accuracy of segmentation depends on the accuracy of registration. The best results are obtained using the GCV_1_ + Vem algorithm. However, the results of GCV_2_ + Vem and GCV_3_ + Vem are also comparable. Detailed summary of Dice coefficient for each considered algorithm is shown in Table 1 in Appendix A.

The results produced by the registration method presented in Papież et al. (2013) show average Dice coefficients varying between 0.86 and 0.92. The joint segmentation and registration methods based on level-set registration algorithms presented in this article give comparable results with the best of 0.96 for GCV_1_ + Vem beating others by around 0.03 on average. Moreover, GCV_1_ + Vem algorithm achieves the highest average Dice measure among all algorithms which do not explicitly account for the sliding discontinuous motion between anatomical structures such as lungs, liver and pleura.

**Fig. 3:**
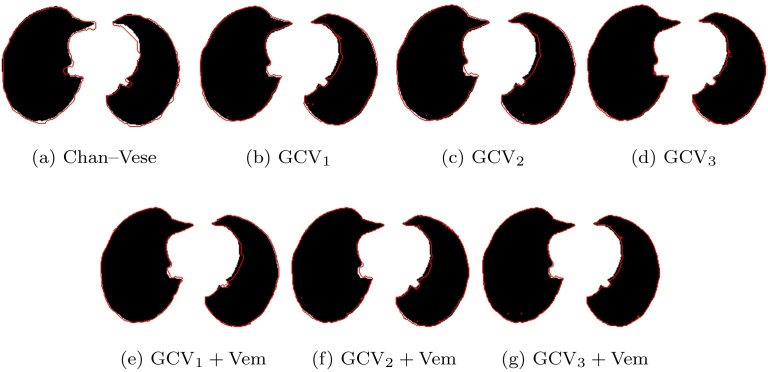
Examples of segmentation results for CT scans of lungs obtained using Chan–Vese algorithm and joint registration–segmentation algorithms introduced in Sections 2 and 3. Images present axial view through segmentations of lung CT images coming from *Dir-Lab* data set. Black regions are segmentations done by experts and red contour surrounds the region segmented with the use of a chosen algorithm. Algorithms GCV_*i*_ + Vem, *i* ∈ {1, 2, 3} are considered with the choice *θ* = 0.5. Chan–Vese segmentation algorithm appears to be the least accurate and is surpassed by the other algorithms for joint registration and segmentation. Incorporating Vemuri's algorithm into Gorthi's framework visibly improves segmentation accuracy (see e–g). These figures confirm results presented in Fig. 2. (For interpretation of the references to color in this figure legend, the reader is referred to the web version of this article.)

In Fig. 3 we present examples of lung segmentations obtained using algorithms discussed above. In algorithms incorporating Vemuri's vector field Eq. (5) into Gorthi's framework, we chose *θ* = 0.5 in vector field Eq. (14). CT scans used in the simulations come from the *Dir-Lab* data base (Castillo et al., 2009). Black regions represent slices through lung segmentations done by experts. Red contours are respective segmentations obtained using considered algorithms. Notice that Fig. 3a–g visually confirm results summarized in Table 2.

### 5.2 Registration accuracy

In the *Dir-Lab* data set each image has been labeled by a number of landmarks annotating anatomical features (Castillo et al., 2009). A registration algorithm, in order to be useful from the medical applications perspective, needs to minimize the distance between points denoting the same point in patient's body before and after the registration procedure. Therefore a commonly used measure for registration evaluation (see Modersitzki, 2009) is the Target Registration Error (TRE) defined as an average Euclidean distance between corresponding points. This means that a set of points **y**_1_, …, **y**_*n*_ ∈ Ω in the target image *I*_*T*_ is specified together with corresponding points **x**_1_, …, **x**_*n*_ ∈ Ω chosen in the source image *I*_*S*_.

$$\text{TRE}(\text{u})=\frac{1}{M}\sum_{i\in \;\text{landmarks}}|{\text{x}}_{i}-({\text{y}}_{i}+\text{u}({\text{y}}_{i}))|,$$

where *M* is the number of landmarks in the images.

**Fig. 4:**
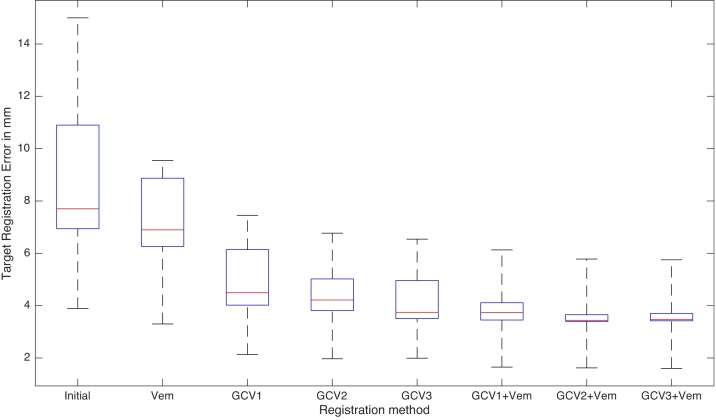
Comparison of the Target Registration Error (in mm) for the registration algorithms. Box edges are 25th and 75th percentiles, central mark represents the median and whiskers extend between maximum and minimum values. Gorthi's algorithm based on prior segmentation gives better results than Vemuri's method. Our method incorporating Vemuri's forces in Gorthi's algorithm improved the accuracy of the methods.

The TRE measure for the each registration algorithm presented in this paper is shown in Fig. 4. On the left-hand side of the figure we present the initial error computed before the registration was performed. The methods evaluated in this test can be divided into three groups: intensity-based registration (Vemuri's algorithm Vemuri et al., 2003) Vem, segmentation-driven registrations (GCV_1_, GCV_2_, GCV_3_) based on Chan–Vese segmentation (Chan et al., 2001), and the proposed joint segmentation and registration methods (GCV_1_ + Vem, GCV_2_ + Vem, and GCV_3_ + Vem). As we can see, the TRE measure decreases for each registration. The highest accuracy in terms of the TRE is achieved by the joint segmentation and registration methods (GCV_1_ + Vem, GCV_2_ + Vem, and GCV_3_ + Vem) with the best TRE = 3.40 mm for GCV_2_ + Vem. The registration algorithms based on segmentation exploiting only the prior knowledge about the position of lungs in the target image yield worse results than the proposed method, but perform well when compared to the classic intensity-based level-set registration Vem. Moreover, a smaller TRE is achieved by the level-set segmentation-based when more regions are selected to drive the registration. This can be explained that considering more regions provides more local information to registration. Our extended algorithms coupling Gorthi's and Vemuri's methods (GCV_1_ + Vem, GCV_2_ + Vem and GCV_3_ + Vem) give not only the most accurate results in terms of TRE but also provide us with the most accurate segmentation methods. Since the landmarks are not used in the algorithm and are used only for evaluation purposes, we consider the joint Gorthi-Vemuri algorithm as the most accurate of all methods presented here. A complete summary of TRE results is presented in Table 2 in Appendix A.

The *Dir-Lab* data set is widely used for registration accuracy evaluation and the TRE computed on this data are known for many methods.^1^ The state-of-the-art registration algorithms involving sliding motion achieve average TRE results varying from 2.76 mm in Delmon et al. (2011), through around 1.5 mm in Papież et al. (2014) to the best known method with the TRE below 1 mm in Rühaak et al. (2013). All mentioned algorithms report better results than the TRE = 3.40 mm achieved by Gor_2_ + Vem. However, the results given by the algorithms presented in this article are comparable with the results obtained by the state-of-the-art *demons* algorithm not modeling sliding motion (Papież et al., 2014). The difference between the input images was used here as a similarity measure to drive registration, however CT volume intensities may change due to lung compression, and so more sophisticated image representation (e.g. multiscale image normals Droske and Rumpf, 2007) could further improve the overall accuracy.

**Fig. 5:**
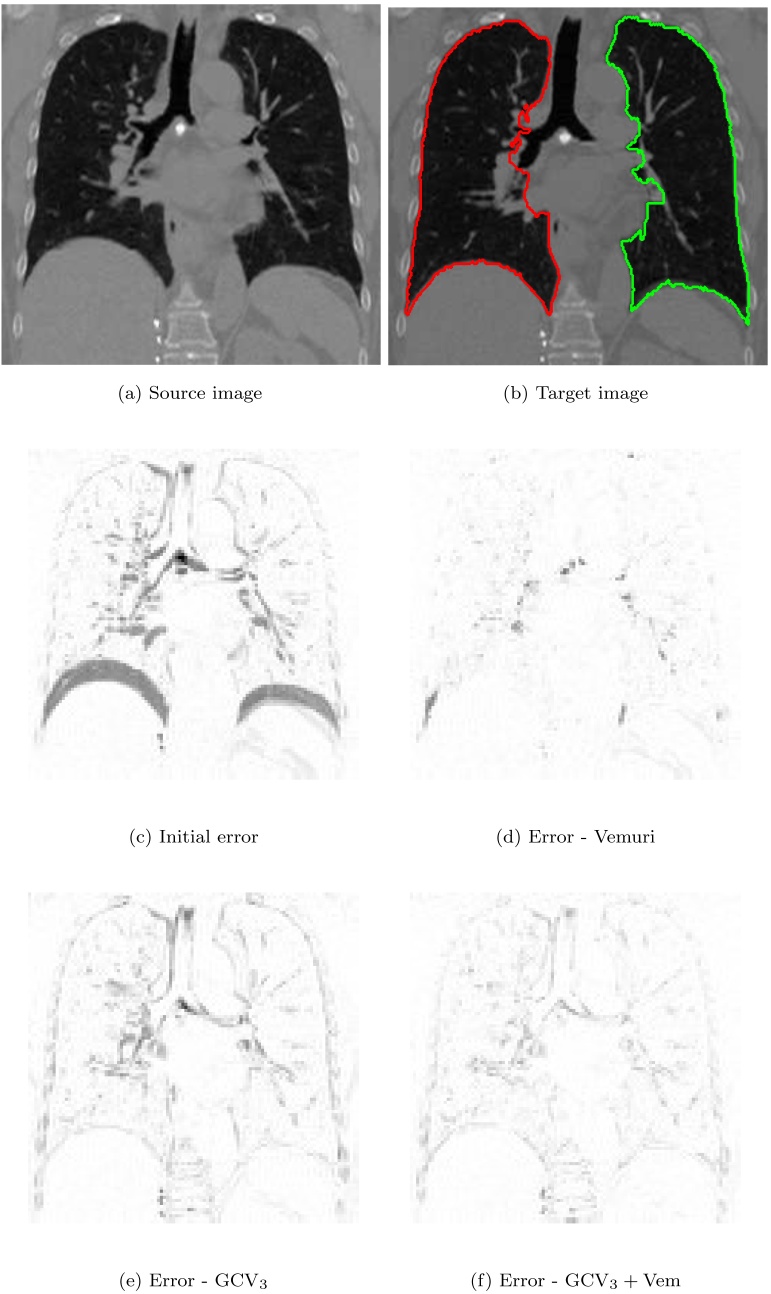
Example of coronal view of 3D registration for CT scans of lungs. The presented method yields noticeable improvement in the alignment, especially in the regions closer to the lung boundaries.

In Fig. 5 examples of registration errors are shown. In the experiments we used CT scans of lungs coming from the *Dir-Lab* data set (Castillo et al., 2009). The source image *I*_*S*_ in Fig. 5a was registered to the target image *I*_*T*_ in Fig. 5b. The initial difference |*I*_*T*_ − *I*_*S*_| is shown in Fig. 5c. The image is dark where the error is large and bright where it is small. We applied three registration algorithms: Vemuri's, modified Gorthi's with the velocity vector field Eq. (13) and their combination with a coupling parameter *θ* = 0.5. Respective errors, normalized by the same factor so they take values between 0 (white) and 1 (black), are presented in Fig. 5d–f. As we can see, the difference between source and target image decreases in the registration process. Notice that the Vemuri's algorithm results in the smallest final difference. However, due to the prior segmentation used in Gorthi's method, the errors shown in Fig. 5e–f are visibly small in the regions occupied by lungs. It is also worth noting that the anatomical shape of lungs is preserved in these images. Note also that all algorithms presented here are less accurate in lung regions close to the ribs. This is because the sliding motion occurring there is not considered in the presented methods.

The number of iteration is tuned to achieve a convergence in terms of TRE. Therefore, the runtime for the presented methods vary remarkably and are as follows. The intensity based registration (Vemuri's algorithm) takes 170 s to reach the stopping criterion (with less than 50 iterations performed). The segmentation-driven algorithms (GCV_1_, GCV_2_, GCV_3_) require significantly more iterations to be performed (about 250) to reach the stopping criterion, and thus their runtime increases to 380 s. The higher number of iteration required to achieve convergence is expected during segmentation-driven registration since only the points lying on contours contribute to the algorithm. The displacement field is diffused to the inside of the segmented structure by the regularization model. The proposed joint segmentation and registration algorithms (GCV_1_ + Vem, GCV_2_ + Vem and GCV_3_ + Vem) require about 160 iterations to converge with the runtime of 248 s. All methods used in this comparison were initialized with the identity transformation as the inhale and exhale volumes included in the Dir-Lab data set come from the same acquisition (there is no need for compensation of patient positioning error using rigid registration).

All algorithms were implemented using MATLAB on a Mac OS with 8 GB of memory and 1.6 GHz Intel Core i5 processor.

## 6 Conclusions

In this article we presented a novel joint segmentation and registration method using the level-set framework. In this framework, we combined the classic Chan–Vese segmentation algorithm with a non-linear intensity-based registration algorithm (Vemuri et al., 2003) using a generalized level-set formulation (Gorthi et al., 2011). It was then extended to the method using several driving forces and furthermore, the presented method was applied to lung CT scans. Compared to the standard registration approaches, our proposed method is able to incorporate a segmentation prior into the cost function with a small computational effort. Furthermore, the accuracy was compared with the state-of-the-art methods for segmentation and registration using the publicly available data set *Dir-Lab* (Castillo et al., 2009). The algorithm presented in this article produces very good segmentation results together with a satisfactory registration accuracy in terms of the TRE. However, the results of our joint segmentation and registration method are still inferior to those achieved by the current state-of-the-art lung registration methods when applied to the *Dir-Lab* data set. This may be due to discontinuous motion between anatomical structures sliding at the chest boundary interfaces, which is not modeled in the presented framework (Papież et al., 2014). Our future work is focused on explicit incorporation of this discontinuous motion at sliding interfaces of lungs into level-set propagation to the registration algorithm proposed here, to improve the overall algorithm's accuracy. Another direction that could be also investigated is a joint partitioning and registration of lung lobes to provide a more realistic description of lung motion (Schmidt-Richberg et al., 2012).

## Figures and Tables

**Table 1:** Comparison of Dice measure for the segmentation algorithms. CV denotes the Chan–Vese algorithm, GCV_*i*_ represents consecutive modifications of Gorthi's algorithm and GCV_*i*_ + Vem are our coupled Gorthi's–Vemuri methods. Our proposed joint registration and segmentation algorithms are more accurate. Gorthi's algorithm gives better results when coupled with Vemuri's method.

100× Dice measure								
Case	Initial	CV	GCV_1_	GCV_2_	GCV_3_	GCV_1_ + Vem	GCV_2_ + Vem	GCV_3_ + Vem
*C1*	91.48	95.27	96.45	95.33	95.98	**97.33**	97.03	97.16
*C2*	91.43	91.32	96.16	96.23	96.21	**97.18**	96.87	96.67
*C3*	89.87	93.44	95.77	95.01	95.30	96.16	96.07	**96.17**
*C4*	88.98	93.60	93.98	94.18	94.22	**94.90**	94.73	94.77
*C5*	90.37	93.16	95.70	95.26	95.23	**96.44**	96.00	96.09
*C6*	85.90	89.16	93.47	93.25	93.53	**94.84**	94.73	94.70
*C7*	89.07	93.76	96.02	95.93	95.91	96.21	96.13	**96.23**
*C8*	89.54	93.68	95.73	95.66	95.62	**96.51**	96.32	96.21
*C9*	91.00	94.32	96.11	96.07	95.98	**96.93**	96.90	96.81
*C10*	90.22	92.18	93.94	94.01	93.96	**96.02**	95.71	95.93

Mean	89.79	92.99	95.33	95.10	95.19	**96.25**	96.05	96.07

**Table 2:** Comparison of the Target Registration Error for the registration algorithms. Gorthi's algorithm based on prior segmentation gives better results than Vemuri's method. Incorporating Vemuri's forces in Gorthi's algorithm improved the accuracy of the methods.

TRE in mm								
Case	Initial	Vemuri	GCV_1_	GCV_2_	GCV_3_	GCV_1_ + Vem	GCV_2_ + Vem	GCV_3_ + Vem
*C1*	3.89	3.30	2.13	1.97	1.99	1.65	1.62	**1.60**
*C2*	4.34	3.85	2.94	2.56	2.35	2.33	**2.07**	2.08
*C3*	6.94	6.26	4.52	4.23	3.86	3.45	**3.41**	3.42
*C4*	9.83	8.74	6.14	4.94	4.46	3.83	**3.40**	3.42
*C5*	7.48	6.83	4.02	3.81	3.50	3.83	**3.44**	3.46
*C6*	10.9	8.87	6.21	5.02	4.96	4.11	**3.65**	3.70
*C7*	11.0	9.13	6.11	5.31	5.02	4.28	**3.69**	3.72
*C8*	15.0	9.55	7.45	6.77	6.54	6.13	5.78	**5.75**
*C9*	7.92	6.34	4.43	4.11	3.61	3.47	**3.43**	3.48
*C10*	7.30	6.96	4.46	4.20	3.62	3.63	**3.50**	3.51

Mean	8.46	6.98	4.84	4.29	3.99	3.67	**3.40**	3.41

## Appendix A

See Table 1, Table 2.

## Footnotes

1 http://www.dir-lab.com/Results.html.
